# Reactive Oxygen Species in Plant Interactions With Aphids

**DOI:** 10.3389/fpls.2021.811105

**Published:** 2022-02-16

**Authors:** Fiona L. Goggin, Hillary D. Fischer

**Affiliations:** Department of Entomology and Plant Pathology, University of Arkansas System Division of Agriculture, Fayetteville, AR, United States

**Keywords:** aphid resistance, chloroplast, hydrogen peroxide, nitric oxide, oxidative burst, peroxisome, R gene, superoxide

## Abstract

Reactive oxygen species (ROS) such as hydrogen peroxide and superoxide are produced in plants in response to many biotic and abiotic stressors, and they can enhance stress adaptation in certain circumstances or mediate symptom development in others. The roles of ROS in plant-pathogen interactions have been extensively studied, but far less is known about their involvement in plant-insect interactions. A growing body of evidence, however, indicates that ROS accumulate in response to aphids, an economically damaging group of phloem-feeding insects. This review will cover the current state of knowledge about when, where, and how ROS accumulate in response to aphids, which salivary effectors modify ROS levels in plants, and how microbial associates influence ROS induction by aphids. We will also explore the potential adaptive significance of intra- and extracellular oxidative responses to aphid infestation in compatible and incompatible interactions and highlight knowledge gaps that deserve further exploration.

## Introduction

### Reactive Oxygen Species in Stress Responses

Regulating the accumulation of reactive oxygen species (ROS) is a critical aspect of how plants adapt to environmental and biotic stresses (Foyer and Noctor, 2016). ROS such as superoxide (O_2_^–^) and hydrogen peroxide (H_2_O_2_) are molecules that contain oxygen and that are highly reactive due to the electron receptivity of this element. They are generated as a byproduct of aerobic metabolic processes such as photosynthesis, and their accumulation is carefully limited by enzymatic and non-enzymatic antioxidants (Apel and Hirt, 2004). Stress may increase ROS levels in plants by limiting antioxidant activities and/or by causing metabolic dysfunctions that increase ROS generation (Ahmad et al., 2010). In addition, ROS can be actively generated in response to stress through genetically programmed enzymatic processes such as O_2_^–^ production by NADPH oxidases or synthesis of photoactivating phytoalexins (Flors and Nonell, 2006). All three of these routes contribute to stress-induced ROS accumulation in plants, and this response represents a double-edged sword (Das and Roychoudhury, 2014). On one hand, ROS can facilitate stress adaptation by activating defensive signaling networks, reprogramming gene expression, modifying cell walls, and in some cases triggering programmed cell death (i.e., the hypersensitive response) to quarantine viruses and other threats (Waszczak et al., 2018). On the other hand, if the timing and magnitude of ROS accumulation are not tightly controlled by the plant’s antioxidant system, ROS can damage the plants’ own membrane lipids, proteins, and DNA, resulting in symptom development rather than defense or acclimation (Demidchik, 2015; Czarnocka and Karpiński, 2018). For example, oxidative stress caused by prolonged, “runaway” ROS accumulation contributes to pathogenesis by necrotrophic pathogens (Barna et al., 2012), and is responsible for much of the damage caused by drought, salinity, heavy metal exposure, and other abiotic stresses (Sharma et al., 2012). In addition to the importance of the timing and magnitude of ROS accumulation, certain studies suggest that the effects of the oxidative response also depend in part upon which ROS are induced and in which cellular compartments they accumulate (Gadjev et al., 2006; Shapiguzov et al., 2012). Therefore, to understand the adaptive significance of ROS accumulation in plants under stress, it is important to know the “who, what, when, and where” of ROS accumulation. In the case of biotic stress, it is also critical to understand the mode of parasitism of the attacker and the ways in which the attacker may manipulate ROS accumulation in its host plant (Barna et al., 2012).

### Regulation of Reactive Oxygen Species Accumulation in the Molecular Arms Race

The cellular redox balance represents an important battleground in the evolutionary arms race between plants and other organisms that utilize them for food and shelter. Although ROS play a role in plant interactions with a diversity of organisms including insects and parasitic plants (e.g., Liu et al., 2010; Bittner et al., 2017; Wada et al., 2019), the majority of studies to date have focused on ROS in plant-pathogen interactions. In response to many phytopathogens, plants generate rapid and transient ROS accumulation at the infection site (i.e., the oxidative burst) that helps limit infection (Lamb and Dixon, 1997). Because of its role in activating programmed cell death, the oxidative burst is especially important in controlling biotrophic or hemibiotrophic pathogens that require living host cells to complete all or part of their life cycle (Glazebrook, 2005). In addition, studies of mutants with impairments in the oxidative burst suggest that at early stages of the infection process, ROS can in some cases contribute to plant defenses against pathogens that live on dead and dying cells (i.e., necrotrophs) (Lehmann et al., 2015). Loss of function of NADPH oxidases in different host plant species has been reported to increase susceptibility to the necrotrophic fungi *Alternaria brassicicola*, *Rhizoctonia solani*, and *Botrytis cinerea* (Pogány et al., 2009; Foley et al., 2013; Li et al., 2015).

Because of the importance of the oxidative burst in plant defense, many pathogens produce effectors that inhibit ROS accumulation in the host plant (Jwa and Hwang, 2017). For example, the powdery mildew *Blumeria graminis* f. sp. *hordei* secretes a catalase to scavenge H_2_O_2_ at the infection site (Zhang et al., 2004), and *Ustilago maydis*, the causal agent of corn smut, decreases ROS production in the host by secreting an inhibitor of peroxidases (Hemetsberger et al., 2012). In contrast to these examples, however, necrotrophic pathogens actively promote ROS accumulation at the infection site during certain phases of the infection process. *Fusicoccum amygdali* for example inhibits catalase activity in host cells (Beffagna and Lutzu, 2007), and *B. cinerea* utilizes its own NADPH oxidases to generate ROS and facilitate infection (Segmüller et al., 2008). The explanation for this apparent contradiction lies in the timing and localization of ROS accumulation; rapid, transient accumulation of ROS at the initial site of infection can inhibit both biotrophs and necrotrophs, whereas slower, more diffuse and sustained ROS accumulation can facilitate necrotrophic infection and symptom development (Pogány et al., 2009; Lehmann et al., 2015).

In addition, ROS may have different consequences depending upon their location within or outside the cell. The oxidative burst associated with pathogen resistance typically involves ROS accumulation in the apoplast, but pathogens and other stresses can also induce ROS generation within cellular compartments such as the chloroplasts and peroxisomes (Kuźniak and Kopczewski, 2020). The same ROS can have different impacts within different organelles; for example, transcript profiles in *Arabidopsis thaliana* lines with altered ROS accumulation suggest that H_2_O_2_ in the chloroplast activates defensive phytoalexin synthesis whereas H_2_O_2_ in the peroxisome activates protein repair and stress acclimation (Sewelam et al., 2014). While intracellular ROS can in some cases have a protective role, in others it can cause oxidative damage and facilitate infection. For example, in the case of *B. cinerea*, persistent ROS levels in the chloroplast potentiate disease development (Rossi et al., 2017), even though early, NADPH-dependent ROS accumulation in the apoplast inhibits infection (Li et al., 2015).

Thus, whether ROS accumulation is adaptive to the plant or to the pathogen depends in part on the pathogen’s lifestyle and its ability to manipulate the host’s oxidative response, as well as the timing and location of ROS accumulation. This conceptual framework is useful to explore the influence of ROS in plant interactions with other biotic stressors.

### Aphids as Plant Parasites

Aphids (Hemiptera:Aphididae) are a large family of sap-feeding herbivorous insects that have many commonalities with plant pathogens (Kaloshian and Walling, 2005). They have coevolved with plants for millions of years (Peccoud et al., 2010), and typically spend their entire lives on their host plants, relying on them for food and shelter. Like plant pathogens, the more than 4,500 extant species of aphids that feed on trees, shrubs, and herbaceous plants display a diversity of lifestyles and modes of plant parasitism (Blackman and Eastop, 2000, 2006). Although most species utilize their slender, piecing-sucking stylets to ingest sap from the phloem sieve elements with occasional bouts of ingestion from the xylem (Tjallingii and Esch, 1993), some species, especially those that feed on woody trunks or stems, feed primarily from parenchyma cells (Pointeau et al., 2012). In addition, while many aphids traverse a primarily intercellular route to reach their feeding sites, sampling cells along the way without causing extensive cellular disruption, certain aphids such as the Russian wheat aphid (*Diuraphis noxia*) also perform frequent, damaging intracellular probes (Pollard, 1973; Tjallingii and Esch, 1993; Saheed et al., 2007).

In part because of this variation in feeding behavior, different aphid species differ in their impacts on host plants. Some species extract nutrients without causing obvious symptoms, others manipulate plant growth and development to induce galls that can support multiple generations of aphids, and yet others such as the Russian wheat aphid or the greenbug (*Schizaphis graminum*) have a more “burnt-earth” approach, causing extensive necrosis and even death of the plant within a relatively short time frame (Miles, 1989; Goggin et al., 2017). Thus, although all aphids are “biotrophs” in the sense that they feed on living tissues, some can, like necrotrophic pathogens, cause extensive necrosis and have relatively short-term residencies on their host plants.

The diversity of symptoms induced by aphids is also related to the diversity of salivary secretions they produce. The composition of aphid saliva varies among different species and different populations and may be influenced by the symbionts they carry (Rao et al., 2013; Chaudhary et al., 2014; Wang et al., 2020). Similar to pathogen effectors, the salivary proteins that aphids secrete during penetration and feeding can facilitate the infestation process on one hand or trigger plant defenses on the other (Bos et al., 2010; Atamian et al., 2013; Elzinga et al., 2014). Moreover, aphid salivary secretions can also contain phytopathogenic viruses because aphids are common virus vectors (Stevens and Lacomme, 2017).

Perhaps because of the similarities between aphids and pathogens and also because of their close associations with bacterial symbionts and viruses, there is considerable overlap between plant responses to pathogens and plant responses to aphids (Kaloshian and Walling, 2005). In particular, many aphid species induce ROS accumulation in their host plants (Tables 1, 2). In addition, plants utilize the same family of nucleotide-binding, leucine-rich repeat resistance genes (R genes) to block parasitism by certain aphids as to combat pathogens such as bacteria, fungi, and viruses (Rossi et al., 1998; Boissot et al., 2016); furthermore, just as virulent pathogen strains have evolved to overcome R genes for disease resistance, virulent aphid isolates have emerged to attack resistant cultivars (Goggin et al., 2001; Sun et al., 2020). These putative gene-for-gene interactions provide useful study systems in which to explore the roles of ROS in plant–aphid interactions. ROS accumulation can be compared in incompatible interactions (i.e., avirulent aphids on resistant host genotypes) versus compatible interactions (avirulent aphids on susceptible plant genotypes, or virulent aphids on either genotype) to identify correlations between ROS and resistance or susceptibility (several examples included in Table 2).

**TABLE 1:** Evidence that aphids induce reactive oxygen species (ROS) accumulation in compatible interactions.

Aphid species	Plant species	Redox response to aphids	Timing of ROS response
*Acyrthosiphon pisum* (Łukaszewicz et al., 2021)	*Pisum sativum* Fabaceae	Increased H_2_O_2_ and O_2_^–^ but did not impact ^⋅^OH. Both superoxide dismutase (SOD) and ascorbate peroxidase (APX) activity increased at later timepoints than ROS accumulation.	Observations at 24, 48, and 72 h after infestation of pea seedlings. While H_2_O_2_ peaked at 24 h and remained higher than controls at 48 h, O_2_^–^ only peaked at 48 h.
*A. pisum* (Woźniak et al., 2019)	*P. sativum*	Increased H_2_O_2_ in the epidermal cells of leaves, with strong accumulation in cell walls. No strong or consistent changes in O_2_^–^. Transiently suppressed SOD activity in roots and leaves; increased semiquinone radicals and peroxidase (POX) activity in foliage; and an increase in a marker of lipid peroxidation in roots and leaves.	Observations at 24, 48, and 72 h, in roots as well as leaves. In response to aphids, H_2_O_2_ in leaves was higher than controls at 24 h, was lower than controls at 48 h, and returned to normal at 72 h.
*A. pisum* (Łukasik et al., 2017)	*P. sativum, Vicia faba*, and *Vicia sativa* Fabaceae	In all three hosts, transient H_2_O_2_ accumulation and initially suppressed catalase (CAT) activity, with a subsequent increase at later time points. APX activity varied among time points and among species.	Observations at 1, 2, 4, 6, 24, 48, and 72 h. H_2_O_2_ increased incrementally from 1 to 6 h, and then decreased incrementally from 6 to 48 h.
*A. pisum* (Mai et al., 2013)	*P. sativum*	Increased H_2_O_2_, O_2_^–^, semiquinone radicals, SOD and CAT activity, and a marker of lipid peroxidation.	Observations at 24, 48, 72, and 96 h after infestation. H_2_O_2_ was significantly elevated only at 24 h, whereas O_2_^–^ steadily increased from 24 to 96 h.
*Aphis craccivora*^1^ (Mai et al., 2017)	*Glycine max* Fabaceae	Increased O_2_^–^ and H_2_O_2_, SOD, CAT, and glutathione peroxidase (GPX) activity, and a marker of lipid peroxidation. ROS, antioxidant activities, and symptom development all increased with increasing infestation levels.	Observations at 6, 12, 24, 48, 72, and 96 h. O_2_^–^ and H_2_O_2_ were elevated at all time points from 6 to 96 h, with O_2_^–^ peaking earlier (∼12 h) than H_2_O_2_ (∼24 h). Higher aphid infestations caused earlier ROS induction than lower infestation levels.
*A. craccivora^1^* (Mai et al., 2016)	*G. max*	Increased H_2_O_2_ and POX activity, with higher infestation levels inducing stronger responses.	Observations at 6, 12, 24, 48, 72, and 96 h. H_2_O_2_ was elevated at 6–24 h, with a maximum at 12 h.
Brachycorynella asparagi^*2^ (Borowiak-Sobkowiak et al., 2016)	*Asparagus officinalis* Asparagaceae	Induced H_2_O_2_, O_2_^–^, and semiquinone radicals in 1- and 2-month old plants, as well as reduced SOD activity in 1-month old plants. Oxidative responses were stronger and more rapid in younger plants, and in response to higher aphid infestation levels. In samples with low ROS induction, H_2_O_2_ was found in the cell walls, whereas in samples with the highest ROS, H_2_O_2_, and O_2_^–^ appeared to be primarily localized to the cytoplasm.	Observations at 24, 48, 72, and 96 h. H_2_O_2_ and O_2_^–^ increased incrementally from 24 to 96 h.
***Cinara tujafilina****^3^* (Durak et al., 2019)	*Thuja orientalis* Cupressaceae	Increased O_2_^–^, SOD, and semiquinone radicals. The early phases of the response were stronger in response to higher aphid densities.	Observations at 24, 48, 72, and 96 h, and at 2 and 4 weeks. The highest O_2_^–^ was observed at 24 h, but the response persisted up to 2 weeks post-infestation.
*Myzus persicae* extract (Prince et al., 2014)	*Arabidopsis thaliana* Brassicaceae	Increased ROS (H_2_O_2_ or O_2_^–^, measured with a luminol assay) was observed in response to an extract consisting of ground aphids in wild-type plants, but not in a mutant with impaired NADPH oxidase activity.	Observations at 0–600 min after treatment with aphid extract. A first ROS peak seen at ∼5–25 min, and a second peak at ∼90–540 min.
*M. persicae* (Ren et al., 2014)	*Nicotiana tabacum* Solanaceae	Efflux of H_2_O_2_ from cells, and increased POD and CAT activities.	Observations at 2 h, 15 h, and 5 days. H_2_O_2_ efflux seen at all time points, with the highest efflux at 15 h.
*M. persicae* (Kerchev et al., 2012)	*Solanum tuberosum* Solanaceae	Increased H_2_O_2_, polyphenols, and POX activity; decreased expression of a peroxisomal CAT and a chloroplastic SOD.	Observations at 8, 24, and 48 h after infestation. H_2_O_2_ increased at 48 h.
***Pemphigus bursarius****^4^* and ***Pemphigus phenax****^4^* (Kmieć and Kot, 2021)	*Populus nigra* Salicaceae	In response to *P. phenax*, H_2_O_2_ was decreased in the tissue surrounding the gall, but not within the gall itself. *P. bursarius* did not impact H_2_O_2_. Fluctuations in guaiacol peroxidase (GPOD) and APX activity as well as a marker of lipid peroxidation were observed in galled tissue and adjacent leaf tissue in response to both aphids.	Observed once when galls were at maturity (approx. 1 month after initial gall formation).
***Pemphigus spyrothecae****^5^* (Kot and Kmieć, 2020)	*P. nigra*	H_2_O_2_ was increased in the gall tissue but decreased in the surrounding leaf tissue compared with intact, non-galled leaves. Decreased GPOD and APX activity as well as a decrease in a marker of lipid peroxidation were also observed.	Observed once when galls reached full maturity (approx. 1 month after initial gall formation), at the time of full gall development.
***Schizaphis graminum****^6^* and *Sitobion avenae*, (Zhang et al., 2019)	*Triticum aestivum* Poaceae	Increased H_2_O_2_ in response to both aphid species, but a stronger response to *S. graminum.*	Observations at 12, 24, 48, and 72 h after infestation. *S. graminum* induced H_2_O_2_ at all time points, w/highest levels at 48 h. The response to *S. avenae* was significantly different from controls only at 48 h.
***S. graminum*** and *Rhopalosiphum padi* (Argandoña et al., 2001)	*Hordeum vulgare* Poaceae	Increased H_2_O_2_ and peroxidase activity.	Observations at 0–35 min. A transient increase in H_2_O_2_ seen at 20 min, returning to baseline by 30 min.
***Tetraneura ulmi****^7^* (Kmieć et al., 2018)	*Ulmus pumila* Ulmaceae	H_2_O_2_ accumulated in the gall and in adjacent leaf tissue with and without evident signs of damage. Decreased CAT, APX, and GPOD activity and increased lipid peroxidation were also observed in galls late in development. CAT, APX, and GPOD also fluctuated in adjacent tissues over the course of gall development.	Observations at 3 time points: early, mid- and late gall development. The highest H_2_O_2_ levels were observed in adjacent undamaged tissue in mid-development.
***Therioaphis trifolii maculata****^8^* (Jiang and Miles, 1993)	*Medicago sativum* Fabaceae	Increased ROS production inferred from discoloration around stylet sheath and cell walls; increased SOD activity; increased O_2_^–^ as inferred from reduction of cytochrome c by leaf extracts.	Observations at 15, 60, and 90 min after infestation. Putative O_2_^–^ accumulation detected at similar levels at all time points.

*Aphid species that cause marked morphological alterations or other severe, diagnostic symptoms are in bold.

*^1^This species is reported to be phytotoxic on amaranth (Bayendi Loudit et al., 2018), cowpea (MacWilliams et al., 2021, preprint) and certain other legumes (Quisenberry and Ni, 2007), but its status on soybean is not to our knowledge reported in published literature.*

*^2^Phytotoxic symptoms described in Borowiak-Sobkowiak et al. (2016).*

*^3^Symptoms described in Durak et al. (2019).*

*^4^Symptoms described by Kmieć and Kot (2021).*

*^5^Symptoms described by Kot and Kmieć (2020).*

*^6^Symptoms described in numerous sources, including Ryan et al. (1990).*

*^7^Symptoms described in Kmieć et al. (2018).*

*^8^Symptoms described in Madhusudhan and Miles (1998) and Goggin et al. (2017).*

**TABLE 2:** Comparison of ROS and NO induction in compatible and incompatible interactions.

Aphid species*	Plant species	Redox response to aphids	Timing of ROS response
*Acyrthosiphon pisum*^£^ (Wang et al., 2020)	*Medicago truncatula* Fabaceae	Increased H_2_O_2_. Compared to uninfected aphids, aphids with the facultative symbiont *Serratia symbiotica* fed more and induced less H_2_O_2_ accumulation.	6 h after infestation or infiltration with aphid saliva.
*Aphis glycines* (Yao et al., 2020)	*Glycine max* Fabaceae	Increased H_2_O_2_ and CAT expression in response to aphids in a susceptible (S) cultivar, but not in two resistant (R) cultivars.	Observations at 24, 48 and 96 h. H_2_O_2_ elevated at 96 h after infestation.
***Diuraphis noxia*^1^** (Moloi and van der Westhuizen, 2014)	*Triticum aestivum* Poaceae	Increased nitric oxide (NO) and nitrate reductase activity in a R cultivar but not a S cultivar.	Observations at 0, 3, 6, 9, 24 and 48 h. NO first detected at 3 h after infestation, and peaked at 9 h.
***D. noxia*** Botha et al., 2014	*T. aestivum*	H_2_O_2_ accumulation observed in infested cultivars with antibiotic (*Dn1*) or antixenotic (*Dn5*) resistance, but not in a S or tolerant (*Dn2*) cultivar. POX induction was also observed in R but not tolerant cultivars.	Observations of H_2_O_2_ at 6 d after infestation.
***D. noxia*** (Berner and van der Westhuizen, 2010)	*T. aestivum*	Increased H_2_O_2_, POD, and SOD in response to aphids in a R but not S genotype; eliminated by an inhibitor of xanthine oxidase in peroxisome.	Observations from 2–48 h after infestation. H_2_O_2_ increased at 8–48 h, with the highest levels ∼16 h and 48 h.
***D. noxia*** (Moloi and Van Der Westhuizen, 2006)	*T. aestivum*	H_2_O_2_, NADPH oxidase activity, and POX increased in response to aphids more strongly in a R than a S genotype. Accumulation was blocked by an inhibitor of NADPH oxidase.	Observations at 3, 6, 9, 12, 24 and 48 h. H_2_O_2_ increased at 3–24 h after infestation and returned to normal at 48 h.
*Macrosiphoniella sanbourni* (Sun et al., 2016)	*Artemisia vulgaris* and *Dendranthema nankingense* Asteraceae	H_2_O_2_, O${}_{2}^{-}$, and a marker of lipid peroxidation all increased in response to aphid infestation in both a susceptible cultivar of chrysanthemum (*D. nankingense*) and a related species that is resistant to aphids (*A. vulgaris*).	Observations at 3, 6, 12, 24, 48, and 168 h. H_2_O_2_ and O${}_{2}^{-}$ were elevated in infested plants at all time points. H_2_O_2_ reached it maximum levels at 6 h in the R species and 72 h in the S species; O${}_{2}^{-}$ reached it maximum levels at 12 h in the R species and 72h in the S species.
*Macrosiphum euphorbiae* (Martinez de Ilarduya et al., 2003)	*Solanum lycopersicum* Solanaceae	Increased H_2_O_2_ in response to aphids in both R and S genotypes.	Observations at 3, 6, 12, and 24 h. H_2_O_2_ detected at 24 h.
***Melanaphis sacchari****^2^* (Pant and Huang, 2021)	*Sorghum bicolor* Poaceae	Increased H_2_O_2_ and expression of genes encoding SOD, APX, CATs, and glutathione *S*-transferases (GSTs) in a R cultivar. Initial decrease in H_2_O_2_ followed by a delayed increase in the S cultivar. For both infested and uninfested treatments, H_2_O_2_ was higher in R than S at all time points.	Observations at 3, 6, 9, and 12 days after infestation. Infestation induced an increase in H_2_O_2_ at all time points in a resistant (R) cultivar, with the highest accumulation at 3 days. In the S cultivar, H_2_O_2_ in infested plants decreased relative to uninfested controls at 3, 6, and 9 days, and increased at 12 days.
***M. sacchari*** (Shao et al., 2019)	*S. bicolor*	Increased H_2_O_2_, and decreased APX and POX activity. H_2_O_2_ induction was more rapid in R than S plants, and higher in R than S at all time points.	Observations at 24, 48, 72, and 96 h. Infestation induced H_2_O_2_ at 24 and 48 h in a R cultivar, and at 48 h in a S cultivar.
*Myzus persicae^¥^* (Guo et al., 2020)	*Nicotiana benthamiana* Solanaceae	Increased H_2_O_2_, and increased expression of a gene encoding NADPH oxidase (*RbohD*) in response to a non-host-adapted aphid lineage with high expression levels of the elicitor cathepsin B3. A host-adapted lineage with lower cathepsin B3 expression did not induce these responses. Transient expression of cathepsin B3 in plant tissues also induced H_2_O_2_.	Observations of responses to aphids at 24 h, and responses to transient *in planta* cathepsin B3 expression at 48 h.
*M. persicae*, *Myzus cerasi*, and *Rhopalosiphum padi* (Jaouannet et al., 2015)	*Arabidopsis thaliana* Brassicaceae	Increased H_2_O_2_, but downregulation of genes associated with ROS metabolism.	Observations at 3, 6, 12, and 24 h. All 3 aphid species induced H_2_O_2_ at 3- and 24 h. The 3 h peak was stronger in response to *M. persicae* than to *R. padi* or *M. cerasi*, whereas the 24 h peak was much stronger in response to *R. padi* or *M. cerasi* than *M. persicae*
*M. persicae^€^* (Lei et al., 2014)	*A. thaliana*	Increased H_2_O_2_ in local but not systemic leaves of the *bik1* mutant, but not in the more susceptible wild-type control Col-0.	Observations at 3, 6, 12, and 24 h. H_2_O_2_ accumulated at all time points in the *bik1* mutant.
*M. persicae* (Sun et al., 2020)	*Capsicum baccatum* Solanaceae	Increased H_2_O_2_ in response to an avirulent aphid population. Virulent aphids did not induce H_2_O_2_, and decreased levels induced by avirulent aphids.	Observations at 72 and 142 h. Significant increase 72 h after infestation w/avirulent aphids; H_2_O_2_ persists even after an additional 72 h without aphids.
*Sitobion avenae* (Czerniewicz et al., 2017)	*Triticosecale* Wittm. Poaceae	H_2_O_2_ was induced by aphid infestation in a R and a S cultivar. O_2_^–^ increased in a R cultivar and decreased in a S cultivar. The R line also showed earlier and stronger induction of phenolic compounds which could have antioxidant activity.	Observations at 24 and 48 h. H_2_O_2_ was elevated at 24 h in a R cultivar and at 48 h in a S cultivar. O_2_^–^ was elevated at both time points in a R cultivar, and decreased in a S cultivar at 48 h.
*S. avenae* and *R. padi* (Łukasik and Goławska, 2019)	*Triticosecale* Wittm.	Increased H_2_O_2_ in response to both aphid species, with a faster, stronger response in a R versus a S cultivar. Higher aphid densities induced higher H_2_O_2_ levels. A marker of lipid peroxidation also increased.	Observations at 6, 24, 48, and 96 h. In a R cultivar, H_2_O_2_ increased in response at all time points, with the highest levels at 24 and 48 h. In S, H_2_O_2_ increased at 24, 48, and 96 h.
*S. avenae* and *R. padi*, (Sytykiewicz, 2016)	*Zea mays* Poaceae	Increased H_2_O_2_ and NADPH oxidase activity in response to both aphid species, with stronger H_2_O_2_ induction in a R cultivar than a S cultivar. Aphid-responsive H_2_O_2_ accumulation was eliminated by an inhibitor of NADPH oxidases.	Observations at 4 and 24 h; H_2_O_2_ induction seen at both time points.
*S. avenae* and *R. padi*, (Sytykiewicz, 2015)	*Zea mays*	Both aphid species induced H_2_O_2_, and higher infestation levels led to higher ROS levels. A highly R cultivar accumulated more H_2_O_2_ than a moderately resistant cultivar, and a moderately R cultivar had more H_2_O_2_ than a susceptible cultivar at both 24 and 48 h. Uninfested R cultivars also had higher basal H_2_O_2_ levels than the S cultivar.	Observations at 2, 4, 8, 24, and 48 h. H_2_O_2_ increased over 2–24 h, and was declining at 48 h.
*S. avenae* and *R. padi*, (Sytykiewicz et al., 2014)	*Zea mays*	Both aphid species induced O_2_^–^ and *GST* gene expression, with higher infestation levels inducing higher ROS levels. O_2_^–^ accumulation was higher in a R than in an S cultivar, and was also higher in response to *R. padi* than *S. avenae.*	Observations at 1, 2, 4, 8, 24, 48, and 72 h. O_2_^–^ reached their maximum at 4 h, but under high infestation levels, remained elevated at 48 h. In response to *R. padi*, O_2_^–^ levels increased more rapidly in the R than the S cultivar.

^£^Although this study does not involve R gene-mediated incompatibility, it compares aphid isolates that differ in their levels of virulence on the host due to the presence or absence of a secondary symbiont. *Aphid species that cause marked morphological alterations or other severe, diagnostic symptoms are in bold. ^¥^This study system is more analogous to non-host plant resistance than to R gene mediated incompatibility. Some populations of M. persicae can colonize tobacco, whereas others cannot. Some sources consider these host races distinct aphid species (tobacco adapted race = Myzus nicotianae; non-host adapted race = M. persicae), although molecular evidence for speciation is lacking (Clements et al., 2000). €This study system does not involve R gene mediated incompatibility, but instead focuses on a null mutation (bik1) that confers aphid resistance.

*^1^Symptoms described in numerous sources, including Burd and Burton (1992).*

*^2^Symptoms described in Singh et al. (2004) and Pant and Huang (2021).*

In summary, plant–aphid interactions display many parallels with plant-pathogen interactions which provide a framework to explore the potential roles of ROS in plant responses to aphid infestation.

### Objectives

Although aphids can induce ROS accumulation in their host plants (Tables 1, 2), the contributions of ROS to plant defense or to symptom development in plant–aphid interactions are not yet well-understood. To begin to address this knowledge gap, this review will synthesize what we know about aphid-responsive ROS accumulation with an emphasis on the “5 Ws”: who, what, when, where, and why. We will consider the factors in aphids that influence ROS accumulation in the host (i.e., “who”), the different RO species that accumulate in infested plants (“what”), and the spatial and temporal dynamics of ROS responses (“when and where”) in compatible and incompatible interactions to explore the adaptive significance (“why”) of oxidative responses to aphid infestation.

## Factors in Aphids that Modify Host Reactive Oxygen Species

There are several possible mechanisms through which aphids may alter ROS levels in plants. The earliest studies of oxidative responses to aphids proposed that they are the result of oxidases in aphid saliva that facilitate the infestation process by detoxifying plant defenses and modifying plant growth (Miles, 1978, 1999). This hypothesis is supported by observations that aphid saliva contains peroxidases and other oxidizing enzymes (Miles, 1999; Carolan et al., 2009, 2011; Nicholson and Puterka, 2014), and that watery saliva collected from the pea aphid (*Acyrthosiphon pisum*) and the spotted alfalfa aphid (*Therioaphis trifolii maculata*) can generate H_2_O_2_ *in vitro* when provided with catechin as a substrate (Madhusudhan and Miles, 1998). More recently, however, a larger number of studies have pursued the hypothesis that ROS are actively generated by the plant in response to aphids as a result of non-self recognition and immune signaling (reviewed in Hogenhout and Bos, 2011). Early evidence for this hypothesis included studies in Arabidopsis that showed that loss of function of plant NADPH oxidases influence aphid population growth (Miller et al., 2009; Jaouannet et al., 2015) and that impaired immune signaling in the plant inhibit ROS production in response to aphid extracts (Prince et al., 2014).

By analogy with plant-pathogen interactions, the recognition process leading to aphid-responsive ROS generation in plants could involve detection of (1) damage-associated molecular patterns (DAMPs) that result from cleavage of plant molecules by the invader; (2) widely-conserved molecular patterns that occur in invading organisms but not in plants; or (3) specific effector proteins found in some but not all species and isolates of invader [reviewed in Jwa and Hwang (2017) and Hou et al. (2019)]. Recently, cathepsin B3 has been identified as an effector protein that varies among *Myzus persicae* lineages and that induces H_2_O_2_ accumulation by physically interacting with a kinase in tobacco that activates NADPH oxidase-dependent ROS generation (Guo et al., 2020). In addition to recognizing aphid effector proteins such as cathepsin B3, plants may also respond to molecular signatures synthesized by aphids’ microbial associates. The GroEL protein present in aphid saliva is synthesized by the obligate endosymbiont *Buchnera aphidicola*, and applying this protein to Arabidopsis foliage induces ROS accumulation and other markers of pathogen-triggered immune responses (Chaudhary et al., 2014). Thus, this microbe-associated molecular pattern (MAMP) could potentially mediate ROS induction by aphids *in vivo*.

Conversely, aphids have also been shown to produce salivary proteins that inhibit ROS induction, including Mp10 and Mp55 from the green peach aphid and ApHRC from pea aphids. Transient expression of Mp10 in plant tissues inhibits the oxidative burst elicited by the common bacterial MAMP flagellin (Bos et al., 2010), and a preprint reports that it also suppresses ROS induction in response to aphid extracts (Drurey et al., 2019). Moreover, ectopic expression of Mp55 or ApHRC in host plants inhibits H_2_O_2_ induction in response to aphid infestation (Elzinga et al., 2014; Wang et al., 2020).

The abundance of aphid salivary proteins that inhibit ROS induction may also be modulated by symbionts. In pea aphids, the facultative symbiont *Serratia symbiotica* promotes expression of the aphid salivary protein ApHRC, which suppresses ROS accumulation in barrel medic (*Medicago truncatula*). Pea aphids without *S. symbiotica* induce ROS, whereas pea aphids with *S. symbiotica* do not; moreover, silencing *ApHRC* expression in aphids with *S. symbiotica* results in ROS induction, and overexpression of *ApHRC* in plant tissues reverses this effect (Wang et al., 2020).

In summary, evidence from multiple systems indicate that (1) plants actively generate ROS in response to aphids; (2) certain aphid effectors trigger the ROS response while other effectors inhibit it; and (3) aphid symbionts can influence ROS induction by producing MAMPs and also by influencing the expression of aphid-encoded effectors.

## Characteristics of the Reactive Oxygen Species Response to Aphids

### Which Reactive Oxygen Species Accumulate in Response to Aphid Infestation?

Studies in a diverse range of experimental systems indicate that aphid infestation induces ROS accumulation (Tables 1, 2). An early study of alfalfa (*Medicago sativum*) challenged with the spotted alfalfa aphid suggested that aphids induce O_2_^–^ based on the ability of leaf extracts to reduce cytochrome c (Jiang and Miles, 1993). Moreover, direct histological detection of O_2_^–^ has since shown that aphid infestation induces this ROS in a gymnosperm (*Thuja orientalis*), as well as in at least four families of angiosperms (Asparagaceae, Asteraceae, Fabaceae, and Poaceae; Tables 1, 2). The majority of studies of ROS generation in response to aphids, however, have focused on hydrogen peroxide (H_2_O_2_), perhaps because the relatively long half-life and lower toxicity of this ROS allow higher titers and longer persistence, facilitating detection. Aphid-responsive H_2_O_2_ accumulation has been demonstrated in at least 22 plant species in eight plant families, including both monocots and dicots (Tables 1, 2). Many of these studies also reported increases in markers of oxidative stress such as lipid peroxidation products, and observed changes in the expression or enzyme activities of pro- and antioxidants (Tables 1, 2). For example, ROS accumulation was often associated with increased peroxidase activity and with an initial decrease in antioxidants such as superoxide dismutase or catalase followed by a later increase in antioxidant activities (e.g., Łukasik et al., 2017; Woźniak et al., 2019). Thus, ROS accumulation is part of a coordinated redox response in the plant. This oxidative response was observed in both resistant and susceptible plants in response to a diversity of aphids with widely varying effects on their hosts, ranging from species that typically do not cause obvious, diagnostic symptoms (e.g., *M. persicae* on Arabidopsis, or *Sitobion avenae* on cereals) to species that cause strong phytotoxic effects such galling (*Tetraneura ulmi, Pemphigus* spp.), leaf rolling (*D. noxia*), malformation of new growth (*Brachycorynella asparagi*), discoloration, necrosis, or even in some cases death of the entire plant (*Melanaphis sacchari, S. graminum*, and *T. trifolii maculata*). Thus, it is clear that aphids can induce O_2_^–^ and H_2_O_2_, and that ROS induction is a widely conserved response in both compatible and incompatible interactions with a diverse array of aphids (Tables 1, 2).

The prevalence of oxidative responses to aphids also suggests that it would be worthwhile to test for the presence of other reactive species beyond H_2_O_2_ and O_2_^–^. Recent advances in the use of luminescent probes and reporter gene systems facilitate the detection of multiple ROS (Ortega-Villasante et al., 2018), and while a recent study did not detect any effects of the pea aphid on hydroxyl radical (^⋅^OH) abundance in pea plants (Łukaszewicz et al., 2021), preliminary data suggests that the green peach aphid may induce singlet oxygen (^1^O_2_) accumulation in Arabidopsis (Fischer, 2021). In addition, Russian wheat aphid infestation on wheat induces accumulation of nitric oxide, a reactive nitrogen species that interacts with ROS to regulate other stress responses (Moloi and van der Westhuizen, 2014; Moloi et al., 2014).

Together, these studies indicate that aphids induce an oxidative response associated with multiple reactive species, and further work is merited to characterize the full range of molecules involved and their impacts on plant defense.

### When Are Reactive Oxygen Species Produced in Response to Aphids?

Our current understanding of the temporal dynamics of ROS responses to aphids is somewhat limited and suggests considerable variation among different plant–aphid interactions. The small number of studies that have measured ROS levels within the first hour after aphid infestation (Argandoña et al., 2001; Xu et al., 2021) or treatment with aphid extracts (Prince et al., 2014) indicate that ROS accumulation can occur within minutes. Moreover, time course studies focused on the early hours of the interaction suggest that this ROS response may have a biphasic pattern similar to the oxidative burst elicited by many plant pathogens (Prince et al., 2014; Jaouannet et al., 2015; Xu et al., 2021). However, most studies have measured ROS at a small number of timepoints between 2 and 96 h after infestation (Tables 1, 2). Of the time series conducted within this range, the majority suggest a transient ROS response that peaks on or before 24 h and then declines, often returning to baseline levels 3–4 days after treatment (Moloi and Van Der Westhuizen, 2006; Moloi and van der Westhuizen, 2014; Ren et al., 2014; Sytykiewicz, 2015; Mai et al., 2016, 2017; Łukasik et al., 2017; Shao et al., 2019; Woźniak et al., 2019). Certain time series studies, however, did not detect significant increases in ROS until well after 24 h (Kerchev et al., 2012; Yao et al., 2020), or observed ROS levels that peaked after 24 h and/or continued to increase steadily in the days following infestation (Borowiak-Sobkowiak et al., 2016; Sun et al., 2016; Zhang et al., 2019). In one of the longest time series studies, O_2_^–^ induction in arborvitae (*T. orientalis*) by the cypress pine aphid (*Cinara tujafilina*) persisted even at 2 weeks after infestation (Durak et al., 2019). Thus, ROS induction by aphids can in some cases be slow and/or quite persistent.

In general, these delayed or persistent ROS responses were observed in compatible interactions, often with aphid species such as the greenbug, the cypress pine aphid, the asparagus aphid (*B. asparagi*), and the poplar spiral gall aphid (*Pemphigus spyrothecae*) that induce marked phytotoxic symptoms (Table 1). Consistent with this pattern, in a comparison of sorghum genotypes that were resistant or susceptible to the phytotoxic sugarcane aphid (*M. sacchari*), aphid challenge on resistant plants induced an earlier oxidative response that peaked at 3 days and then declined, whereas H_2_O_2_ levels in the susceptible cultivar initially decreased in response to aphids and then increased at 12 days post-infestation, when extensive aphid damage was present (Pant and Huang, 2021). However, heightened H_2_O_2_ levels have also been observed in an aphid-resistant chili pepper cultivar 6 days after challenge with avirulent green peach aphids even when aphids were removed 3 days prior (Sun et al., 2020); furthermore, a sorghum genotype resistant to the sugarcane aphid was reported to have constitutively high H_2_O_2_ in comparison to a susceptible genotype even in the absence of infestation (Shao et al., 2019). Therefore, certain incompatible interactions also appear to involve long-term ROS accumulation. In addition, the limited number of studies that have utilized more than one probe for ROS detection indicate that H_2_O_2_ and O_2_^–^ can in some cases differ from one another in their onset and persistence (Mai et al., 2013, 2017; Sun et al., 2016; Czerniewicz et al., 2017), and that the timing of the oxidative response to aphids also varies among subcellular compartments (Xu et al., 2021).

In summary, aphid-responsive ROS can occur within minutes and persist for days or even weeks after infestation. Resistance is typically associated with rapid ROS responses and persistent ROS accumulation is often associated with aphid damage; however, exceptions exist to these generalizations, and additional, more detailed studies on the temporal and spatial dynamics of ROS are needed in order to establish correlations with defense mechanisms or symptom development. The *in planta* expression of luminescent probes that can reversibly respond to ROS is a particularly promising approach to study the temporal dynamics of the oxidative response to aphids because it can allow continuous, non-destructive monitoring of ROS accumulation in live tissues (Xu et al., 2021).

### Where and How Are Reactive Oxygen Species Generated in Response to Aphids?

Our current understanding of the spatial dynamics of ROS induction by aphids is even more limited than our knowledge of the temporal patterns of this response. Although the saliva of certain aphids has been reported to translocate within the plant (Madhusudhan and Miles, 1998) and other biotic and abiotic stresses have been shown to trigger systemic ROS signaling (Miller et al., 2009), few studies have explored the potential for long-distance oxidative responses to aphids. Lei et al. (2014) compared H_2_O_2_ levels in adjacent Arabidopsis leaves with and without green peach aphid infestation and saw ROS accumulation only in infested leaves. However, Woźniak et al. (2019) reported that aphid infestation on asparagus foliage induced lipid peroxidation and modified superoxide dismutase and peroxidase activities in roots as well as leaves, suggesting that aphid infestation can have systemic effects on plant redox balance. Thus, further studies are warranted to characterize the spatial patterns of aphid-responsive ROS induction throughout the host plant.

Additional studies of ROS localization are also needed at the cellular and subcellular levels because current evidence suggests that aphid infestations induce ROS accumulation both extra- and intracellularly, via multiple biosynthetic mechanisms. In aphid-infested pea foliage, H_2_O_2_ was observed in the apoplast in association with the cell walls of epidermal cells (Woźniak et al., 2019). Potentially, the extracellular H_2_O_2_ pool could result from generation of O_2_^–^ by plasma membrane-associated NADPH oxidases and conversion of O_2_^–^ to H_2_O_2_ by superoxide dismutases. This mechanism of ROS generation drives the oxidative burst associated with many forms of pathogen resistance in plants (Wang et al., 2018). Experimental inhibition of NADPH oxidases has been reported to block aphid-responsive H_2_O_2_ generation in wheat (*Triticum aestivum*), Arabidopsis, and corn (*Zea mays*), supporting the hypothesis that NADPH oxidases mediate extracellular ROS accumulation in response to aphids (Moloi and Van Der Westhuizen, 2006; Prince et al., 2014; Jaouannet et al., 2015; Sytykiewicz, 2016). Interestingly, aphid-responsive ROS accumulation in Arabidopsis appears to depend more heavily on the NADPH oxidase RbohF than on its homolog RbohD, whereas RbohD is primarily responsible for pathogen-inducible ROS accumulation (Jaouannet et al., 2015). Thus, different NADPH oxidases may be specialized to respond to different stresses. H_2_O_2_ in the apoplast also could be generated by cell wall peroxidases, as is seen in certain plant-pathogen interactions (O’Brien et al., 2012); notably, multiple studies have observed increases in peroxidase activity in response to aphid infestation (Table 1).

In addition to arising in the apoplast, H_2_O_2_ could originate intracellularly and be exported from the cell; consistent with this, *M. persicae* infestation induces sustained H_2_O_2_ efflux in tobacco (*Nicotiana tabacum*) (Ren et al., 2014). Although few studies have examined the subcellular localization of oxidative responses to aphids, we have recently shown that *M. persicae* infestation on Arabidopsis causes rapid and sustained oxidation in the cytosol, peroxisomes, and chloroplasts (Fischer, 2021; Xu et al., 2021). The timing of these intracellular oxidative responses suggests that they are distinct from apoplastic ROS accumulation (Xu et al., 2021). *M. persicae* infestation on potato (*S. tuberosum*) also upregulates expression of a peroxisomal catalase and a chloroplastic SOD, suggesting an impact of aphids on intracellular redox balance (Kerchev et al., 2012). Moreover, an inhibitor of peroxisomal xanthine oxidase blocks H_2_O_2_ induction by *D. noxia* in wheat (Berner and van der Westhuizen, 2010).

Together, these studies suggest that both extracellular and intracellular sources of ROS contribute to the oxidative response to aphid infestation. Consistent with this hypothesis, H_2_O_2_ was detected in the cell walls of aphid-infested asparagus (*Asparagus officinalis*) at early time points and low infestation levels, whereas it appeared to have a primarily cytosolic location at later time points and in samples that had higher ROS levels and more extensive symptom development due to the use of higher infestation levels or more susceptible life stages of the host plant (Woźniak et al., 2019). Similarly, our observations in Arabidopsis suggest that apoplastic ROS accumulation is limited to the early hours of the response, whereas intracellular ROS generation in the peroxisome and cytosol may be more persistent (Xu et al., 2021).

In short, it appears that different extra- and intercellular compartments contribute to different phases of the oxidative response, and further studies are warranted to investigate how these different sources of ROS impact the outcome of the plant–aphid interaction.

### What Causes Variation in Reactive Oxygen Species Generation in Response to Aphids?

Although ROS induction has been observed in a wide array of plant–aphid interactions, this response also displays considerable variability. The onset and duration of ROS accumulation varies widely in different studies (Tables 1, 2). Moreover, not all studies that have measured ROS have detected an increase in response to aphids; for example, using 3,3′-diaminobenzidine (DAB) staining, Kuśnierczyk et al. (2008) could not detect H_2_O_2_ accumulation in Arabidopsis in response to the cabbage aphid, *Brevicoryne brassicae*, even though they observed induction of numerous genes related to H_2_O_2_ signaling. Different ROS detection methods vary in how sensitive and quantitative they are, which can contribute to study-to-study variation (Queval et al., 2008). Study-to-study variation likely also reflects a variety of biological sources of variability. Side-by-side comparisons have revealed that the magnitude of ROS induction varies depending upon the aphid species (Sytykiewicz et al., 2014; Jaouannet et al., 2015; Zhang et al., 2019; Kmieć and Kot, 2021), the aphid biotype (Guo et al., 2020; Sun et al., 2020), the plant cultivar (Table 2), plant age (Borowiak-Sobkowiak et al., 2016), location of the aphid infestation on the plant (Kmieć and Kot, 2021), and aspects of the experimental design such as infestation levels and timing of measurement (Borowiak-Sobkowiak et al., 2016; Mai et al., 2017; Durak et al., 2019; Łukasik and Goławska, 2019). For example, when different infestation levels were compared on soybean, asparagus, and wheat, ROS levels peaked more rapidly in plants challenged with higher aphid densities (Borowiak-Sobkowiak et al., 2016; Mai et al., 2017; Durak et al., 2019; Łukasik and Goławska, 2019).

Most of all, the scattered and asynchronous nature of aphid feeding sites poses special experimental challenges. In comparison to bacteria or other pathogens that can be infiltrated into leaves at high concentrations, even very high numbers of aphids establish a fairly low concentration of feeding sites on infested tissues, resulting in far fewer sites of cellular interaction. Moreover, these cellular interactions are highly asynchronous because aphids do not all initiate feeding at the same time or feed for the same duration, and can initiate new feeding sites at any time. As a result, investigators have limited experimental control over the density of feeding sites and the stage(s) of the interaction that are being sampled. This can limit signal strength, increase random sample-to-sample variation, and make it difficult to pinpoint the timing of transient responses.

We propose that a finer-scale kinetic analysis of aphid-responsive ROS accumulation is warranted, especially since the timing and localization of oxidative responses impact their potential contributions to defense. Highly sensitive luminescent probes for ROS detection could potentially be applied to study the spatial and temporal dynamics of the ROS response at individual feeding sites, and could open new avenues of research in host-plant resistance to aphids.

## Why? Effects of Reactive Oxygen Species Accumulation on Resistance or Susceptibility to Aphids

### Correlations Between Reactive Oxygen Species and Resistance

One approach to assess the functional significance of ROS in plant–aphid interactions is to compare the timing and magnitude of ROS accumulation in plant–aphid combinations with varying degrees of compatibility (Table 2). To this end, numerous studies have examined compatible and incompatible interactions governed by resistance genes in host plants and effector genes in aphids; in addition, a smaller number of studies have examined non-host resistance (Jaouannet et al., 2015) and gain-of-function resistance resulting from mutagenesis (Lei et al., 2014). Of 14 studies that compared ROS accumulation in aphid-resistant and aphid-susceptible plant genotypes, 12 of these studies observed stronger and/or faster ROS accumulation on the resistant plants, with ROS accumulation in some cases being entirely absent in the susceptible varieties (Table 2). For example, in wheat challenged with the Russian wheat aphid, extensive H_2_O_2_ accumulation was observed in resistant but not in tolerant or susceptible genotypes (Botha et al., 2014). This oxidative response occurred in both antibiotic and antixenotic forms of resistance, but was stronger in plants with the *Dn1* gene for antibiosis, possibly because *Dn1* mediates a strong hypersensitive response (Botha et al., 2014). This and other studies in Table 2 suggest that ROS may contribute to multiple resistance pathways, and highlight the need for more mechanistic studies to determine how ROS impact plant defenses against aphids.

Other evidence for a correlation between ROS and plant defense comes from studies of aphid biotypes, host races, or species that vary in their ability to colonize the same host. Avirulent green peach aphids induced persistent H_2_O_2_ accumulation in a resistant cultivar of pepper (*Capsicum baccatum*), but a virulent biotype did not, and could reverse prior ROS induction by avirulent aphids (Sun et al., 2020). Moreover, in comparison to a lineage of the green peach aphid that could colonize *Nicotiana benthamiana*, a lineage that was not adapted to this host induced significantly higher H_2_O_2_ levels in tobacco and expressed higher levels of the elicitor cathepsin B3 (Guo et al., 2020). Similarly, aphid species that could not colonize Arabidopsis (*Rhopalosiphum padi*) or that colonized it only weakly (*Myzus cerasi*) induced stronger H_2_O_2_ accumulation at 24 h than *M. persicae*, which is well-adapted to Arabidopsis (Jaouannet et al., 2015). Interestingly, at 3 h *M. persicae* actually induced stronger H_2_O_2_ accumulation than *R. padi* or *M. cerasi*, but it also more strongly downregulated genes associated with ROS metabolism.

Together, these studies suggest that strong ROS responses within the first 24 h are correlated with incompatibility, and that the ability of aphids to suppress these ROS responses promote compatibility. However, there are some exceptions to this generalization. The soybean aphid induced ROS accumulation at 96 h in a susceptible soybean cultivar but not in a resistant genotype, indicating that ROS accumulation is not necessarily associated with all forms of aphid resistance (Yao et al., 2020). Furthermore, a direct comparison of the phytotoxic greenbug *S. graminum* and a non-phytotoxic species (the English grain aphid, *S. avenae*) on susceptible winter wheat (*T. aestivum*) revealed earlier, stronger, and more persistent ROS accumulation in response to the phytotoxic species, suggesting a correlation between ROS generation and symptom development (Zhang et al., 2019). Prolonged ROS induction by other phytotoxic species such as the cypress pine aphid and the asparagus aphid was also accompanied by extensive symptom development (Borowiak-Sobkowiak et al., 2016; Durak et al., 2019).

To summarize, these studies indicate that rapid ROS induction is often correlated with aphid resistance, but that persistent ROS accumulation may in some cases cause oxidative stress, contributing to symptom development in some compatible interactions.

### Association With Nitric Oxide Induction

To add support to the idea that plant defenses against aphids are associated with rapid redox reactions, there is also evidence that the reactive nitrogen species nitric oxide (NO) plays a role in plant–aphid interactions (Arnaiz et al., 2021). Pea aphids induce extracellular NO accumulation in pea in the first 48 h of the interaction in conjunction with induction of defensive hormones including jasmonates, salicylic acid, and ethylene (Mai et al., 2014). The Russian wheat aphid induces NO accumulation and nitrate reductase activity within 3 h in a resistant wheat cultivar but not in a near-isogenic susceptible cultivar (Moloi and van der Westhuizen, 2014). Exogenous treatments with a NO donor, sodium nitroprusside (SNP) reduces the intrinsic rate of increase of the Russian wheat aphid on wheat (Moloi et al., 2014) and decreases the feeding behavior and fecundity of the pea aphid on pea (Woźniak et al., 2017). Together, these results suggest that NO contributes to plant defenses against aphids. Furthermore, Russian wheat aphids also trigger accumulation of peroxynitrite, a product generated when NO reacts with O_2_^–^ radicals (Moloi et al., 2014). This supports the hypothesis that NO interacts with ROS to regulate plant responses to aphids. ROS can induce NO synthesis, and reactive oxygen and nitrogen species react chemically and interact synergistically to promote many plant responses to biotic and abiotic stress (Wang et al., 2013; Farnese et al., 2016). For example, the interaction between NO and H_2_O_2_ mediates the hypersensitive response to pathogens and also stomatal closure in response to drought (Delledonne et al., 2001; Bright et al., 2006). Whereas ROS induce NO synthesis, NO can hamper further ROS accumulation by inhibiting NADPH oxidase activity and by promoting antioxidant activities (Farnese et al., 2016); for example, in Arabidopsis, NO enhances the activity of a cytosolic ascorbate peroxidase by *S*-nitrosylation (Yang et al., 2015). In this way NO may help fine-tune the timing of ROS responses and limit oxidative damage.

In summary, there is evidence from pea and wheat that NO contributes to plant defenses against aphids, and may interact with ROS to modulate the oxidative response.

### Effects of Aphid Effectors on Aphid Performance

Since several studies have identified effectors in aphid saliva that promote or inhibit ROS accumulation in the host plant, another approach to explore the adaptive significance of the ROS response is to determine how modifying effector expression impacts aphid performance. RNA interference (RNAi) to suppress expression of the ROS elicitor cathepsin B3 increases feeding by a non-host adapted green peach aphid lineage on *N. benthamiana* (Guo et al., 2020). Similarly, ectopic expression of the symbiont-derived ROS elicitor GroEL in plants reduces the fecundity of the green peach aphid and also the potato aphid, *Macrosiphum euphorbiae* (Chaudhary et al., 2014). *In planta* expression of Mp55, a salivary inhibitor of ROS induction, increases the attraction of the green peach aphid to transgenic Arabidopsis plants in choice tests (Elzinga et al., 2014). RNAi of another ROS inhibitor, Mp10, decreases green peach aphid numbers; moreover, a preprint reports that Mp10 is conserved in other plant-feeding Hemipterans such as whiteflies, psyllids, and leafhoppers, but not in carnivorous Hemipterans, suggesting that it is important to the plant-parasitic lifestyle (Drurey et al., 2019). It is important to remember that each of these salivary proteins likely has multiple impacts on plants and aphids, and so their effects on aphid fitness are not necessarily due exclusively to their impacts on ROS accumulation. Nonetheless, when taken together, the studies to date suggest that that ROS induction limits aphid feeding and reproduction, and that salivary effectors that hamper ROS induction benefit aphid fitness.

### Effects of Artificial Manipulation of Reactive Oxygen Species Accumulation on Aphid Performance

The adaptive significance of ROS in plant–aphid interactions can also be investigated by experimental manipulation of ROS levels, either through direct modification of the host plant or through microbes that influence ROS levels in plants. Several studies that artificially manipulate ROS accumulation in host plants support the hypothesis that ROS play a role in limiting aphid infestations. In Arabidopsis, mutations that impair NADPH oxidase activity increase the survival of *R. padi*, a species that cannot reproduce on Arabidopsis, and these mutations also enhance the population growth of the green peach aphid and a related species (*M. cerasi*) that normally has limited ability to colonize this plant (Miller et al., 2009; Jaouannet et al., 2015). Conversely, a gain-of-function mutation in Arabidopsis that results in constitutively higher ROS accumulation decreases infestations by the green peach aphid and the cabbage aphid (*B. brassicae*) (Chen et al., 2014). In potato, a chemical treatment that promotes accumulation of the antioxidant ascorbic acid also increases green peach aphid numbers (Kerchev et al., 2012).

In addition, it appears that microbes associated with aphids or plants can hurt or help aphid fitness depending upon whether they enhance or suppress the ROS response in host plants. When the green peach aphid transmits Cucumber Mosaic Virus (CMV) to tobacco, the virus induces H_2_O_2_ accumulation in the host plant and reduces aphid feeding; the effects of CMV on aphid feeding can be eliminated by deleting the viral protein that triggers ROS induction or by suppressing NADPH oxidase activity in the plant (Guo et al., 2019). Therefore, the inhibitory effects of CMV on aphid feeding appear to be due to induction by CMV of NADPH oxidase-dependent H_2_O_2_ generation in the host plant. Conversely, the facultative aphid symbiont *S. symbiotica* enhances pea aphid feeding on barrel medic by promoting expression of a salivary protein (ApHRC) that limits ROS induction; silencing this protein increases ROS induction and decreases aphid feeding (Wang et al., 2020). Treating plants with certain plant growth-promoting rhizobacteria also causes a faster, stronger oxidative response to aphids and decreases aphid population growth (Rashid et al., 2017; Veselova et al., 2019); this finding is particularly exciting because it suggests a possible means of exploiting ROS for crop protection. Thus, manipulation of ROS levels in multiple plant–aphid interactions implicate ROS in plant defenses against aphids. However, not all such studies support this conclusion. Population growth of the green peach aphid on Arabidopsis was inhibited by a transgene that increases the antioxidant ascorbic acid in the apoplast (Rasool et al., 2017), and by loss of function of *OXI1*, a downstream component of H_2_O_2_ signaling (Shoala et al., 2018). Loss of function of catalase 2, which converts O_2_^–^ to H_2_O_2_ in the chloroplast, also decreases green peach aphid fecundity on Arabidopsis, although it is unclear whether this effect is due to decreased H_2_O_2_, increased O_2_^–^, or other possible effects of catalase activity (Rasool et al., 2019). A mutation (*cpl1*) that decreased the sensitivity of Arabidopsis to O_2_^–^ also decreased plant symptom development after prolonged (14 days), heavy infestations of the green peach aphid (Thatcher et al., 2018).

Together, these reports suggest that while ROS contribute to defense in many plant–aphid interactions, aspects of the oxidative response can also facilitate aphid infestation and/or promote feeding damage in certain compatible interactions.

### Challenges in Studying the Adaptive Significance of Reactive Oxygen Species

When interpreting experiments to manipulate *in vivo* ROS accumulation, is important to remember that pro- and antioxidants in plants are intertwined in a complex redox signaling system (Noctor et al., 2016). For example, as noted in Tables 1, 2, ROS responses to aphid feeding are often accompanied by changes in the expression levels or activity of multiple anti- or pro-oxidant enzymes. Experiments that modify any single component in the plant redox system may have complex pleiotropic effects, or conversely, may simply be outweighed by other components with overlapping functions. Moreover, the tools that are most commonly available to manipulate ROS (chemical inhibitors, null mutations, overexpression lines, etc.) have limited capacity to modulate the important temporal and spatial dynamics of the redox response. Thus, although functional genomics and other manipulative experimental approaches have advanced our understanding of plant–aphid interactions immensely, we must exercise caution in interpreting these experiments. In the future, as our understanding of individual pro- and antioxidant systems increases, we hope that this knowledge will be integrated to provide a more comprehensive systems approach to redox responses to aphid challenge.

## Discussion

### Summary

**FIGURE 1:**
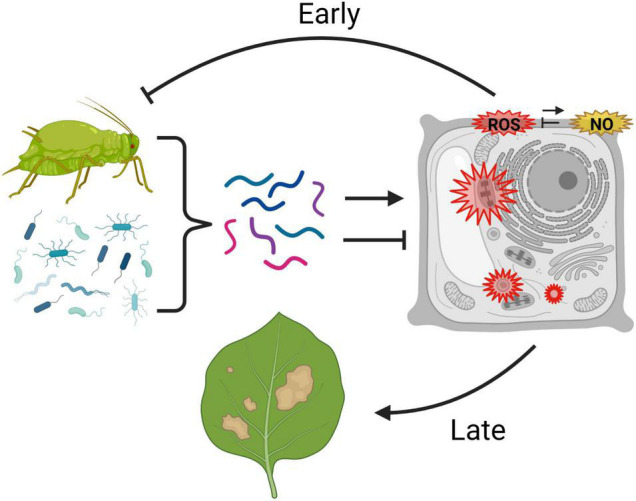
Possible model for the roles of reactive oxygen species (ROS) and nitric oxide (NO) in plant–aphid interactions. Aphids and their microbial associates produce a combination of effectors (represented by multicolored lines) that can promote or inhibit an oxidative response in the host plant. Aphid-responsive reactive oxygen species production (ROS, in red) is known to occur in the apoplast, cytosol, chloroplast, and peroxisomes, and may potentially be modulated by nitric oxide (NO, in yellow), which also accumulates in the apoplast in response to aphids. Rapid, early ROS responses are thought to contribute to certain forms of aphid resistance, whereas delayed, persistent ROS accumulation, particularly intracellularly, may contribute to symptom development in response to infestation. This graphic was created using BioRender.

Over 40 years of research on a wide range of plant taxa have demonstrated that plants produce O_2_^–^, H_2_O_2_, and NO in response to effectors from aphids and their symbionts. Moreover, oxidative responses are common in both compatible and incompatible interactions with a wide range of aphids, including asymptomatic as well as phytotoxic species. Figure 1 summarizes a potential model for the roles of ROS in plant–aphid interactions. Oxidative responses to aphids involve both apoplastic and intracellular sources of ROS, and the timing of ROS accumulation can vary from minutes to days. Although exceptions to this generalization exist, ROS accumulation in incompatible interactions is most often rapid and transient, whereas delayed, persistent ROS accumulation is most often observed in compatible interactions with phytotoxic species. In the last 10 years, advances in genomics have enabled the identification of factors in aphids and their associated microbes that influence the oxidative response in host plants. Certain effectors that induce ROS can reduce aphid performance on plants, whereas other effectors that hamper ROS induction appear to facilitate the infestation process. Likewise, several treatments that compromise the plants’ ability to generate ROS, such as loss of function of NADPH oxidases, have been shown to increase susceptibility to aphids. These studies strongly suggest that ROS promote resistance in many incompatible interactions and also contribute to basal defenses that limit the extent of infestation in compatible interactions. However, evidence also exists for a role of certain components of the ROS response, such as OXI-signaling, in susceptibility and symptom development. Potentially, the oxidative response may mediate resistance in some aphid-host combinations but contribute to susceptibility in others. Another explanation that is not mutually exclusive is that even within a single plant–aphid interaction, different phases and components of the oxidative response may have differing adaptive significance. In particular, the identity of the RO species involved, the subcellular compartment in which they originate, and the speed, magnitude, and persistence of accumulation may all influence whether ROS contribute to aphid resistance or susceptibility. Further work is needed to dissect the impacts of the different phases and components of the plant redox response on levels of resistance or susceptibility to aphids.

### Future Directions

New approaches to characterize and manipulate the timing and localization of specific ROS are needed for a more complete understanding of oxidative responses to aphid infestation. In particular, further studies are warranted to: (1) investigate the adaptive significance of ROS production in the chloroplast and other intracellular sites compared with apoplastic ROS; (2) explore the role of NO in modulating the ROS response; (3) identify the downstream effects of ROS that may mediate resistance or susceptibility, such as the potential impacts of ROS on cell walls, membrane lipids, post-translational protein modification, and global gene expression; and (4) integrate our knowledge of individual pro- and antioxidants in the plant into a more comprehensive systems-view of redox responses to aphid challenge. Research in this area is opening new horizons in agroecology; for example, it is helping to explain how and why components of the microbial community such as aphid symbionts, viruses, and plant rhizobacteria can influence plant susceptibility to aphids (Rashid et al., 2017; Guo et al., 2019; Wang et al., 2020). This research also has exciting applications for pest management such as the potential use of rhizobacteria as biocontrol agents to induce ROS-mediated aphid resistance (Veselova et al., 2019). Most of all, this field of study is important to understand plant adaptation to stresses in a changing world. Plant interactions with aphids are impacted by drought, extreme temperatures, and other harsh environmental conditions, and the plant’s redox regulation, sensing, and signaling system mediates these interactive effects (Foyer et al., 2016). Indeed, this system is a nexus linking abiotic and biotic stress responses, and contributes to induced resistance, induced susceptibility, stress priming, and long-term memory (Carmody et al., 2016). Therefore, in the face of increasingly extreme and variable climactic conditions, understanding plant redox responses to aphids is critical to the future of crop protection.

## Author Contributions

FG and HF conceived and wrote this review article. Both authors contributed to the article and approved the submitted version.

## Conflict of Interest

The authors declare that the research was conducted in the absence of any commercial or financial relationships that could be construed as a potential conflict of interest.

## Publisher’s Note

All claims expressed in this article are solely those of the authors and do not necessarily represent those of their affiliated organizations, or those of the publisher, the editors and the reviewers. Any product that may be evaluated in this article, or claim that may be made by its manufacturer, is not guaranteed or endorsed by the publisher.

## References

[B1] Ahmad P. Jaleel C. A. Salem M. A. Nabi G. Sharma S. (2010). Roles of enzymatic and nonenzymatic antioxidants in plants during abiotic stress. *Crit. Rev. Biotech.* 30 161–175. 10.3109/07388550903524243 20214435

[B2] Apel K. Hirt H. (2004). Reactive oxygen species: metabolism, oxidative stress, and signal transduction. *Annu. Rev. Plant Biol.* 55 373–399. 10.1146/annurev.arplant.55.031903.141701 15377225

[B3] Argandoña V. H. Chaman M. Cardemil L. Muñoz O. Zúñiga G. E. Corcuera L. J. (2001). Ethylene production and peroxidase activity in aphid-infested barley. *J. Chem. Ecol.* 27 53–68. 10.1023/A:1005615932694 11382067

[B4] Arnaiz A. Rosa-Diaz I. Romero-Puertas M. C. Sandalio L. M. Diaz I. (2021). Nitric oxide, an essential intermediate in the plant–herbivore interaction. *Front. Plant Sci.* 11:2029. 10.3389/fpls.2020.620086 33488661 PMC7819962

[B5] Atamian H. S. Chaudhary R. Cin V. D. Girke T. Kaloshian I. (2013). *In planta* expression or delivery of potato aphid *Macrosiphum euphorbiae* effectors Me10 and Me23 enhances aphid fecundity. *Mol. Plant Microbe Interact.* 26 67–74. 10.1094/MPMI-06-12-0144-FI 23194342

[B6] Barna B. Fodor J. Harrach B. D. Pogány M. Király Z. (2012). The Janus face of reactive oxygen species in resistance and susceptibility of plants to necrotrophic and biotrophic pathogens. *Plant Physiol. Biochem.* 59 37–43. 10.1016/j.plaphy.2012.01.014 22321616

[B7] Bayendi Loudit S. M. Bauwens J. Francis F. (2018). Cowpea aphid–plant interactions: endosymbionts and related salivary protein patterns. *Entomol. Exp. Appl.* 166 460–473. 10.1111/eea.12687

[B8] Beffagna N. Lutzu I. (2007). Inhibition of catalase activity as an early response of *Arabidopsis thaliana* cultured cells to the phytotoxin fusicoccin. *J. Exp. Bot.* 58 4183–4194. 10.1093/jxb/erm275 18039736

[B9] Berner J. M. van der Westhuizen A. J. (2010). Inhibition of xanthine oxidase activity results in the inhibition of Russian wheat aphid-induced defense enzymes. *J. Chem. Ecol.* 36 1375–1380. 10.1007/s10886-010-9879-y 21086025

[B10] Bittner N. Trauer-Kizilelma U. Hilker M. (2017). Early plant defence against insect attack: involvement of reactive oxygen species in plant responses to insect egg deposition. *Planta* 245 993–1007. 10.1007/s00425-017-2654-3 28175992

[B11] Blackman R. L. Eastop V. F. (2000). *Aphids On the World’s Crops: An Identification and Information Guide.* Hoboken, NJ: John Wiley & Sons.

[B12] Blackman R. L. Eastop V. F. (2006). *Aphids on the World’s Herbaceous Plants and Shrubs.* New York, NY: John Wiley & Sons, Inc.

[B13] Boissot N. Schoeny A. Vanlerberghe-Masutti F. (2016). *Vat* an amazing gene conferring resistance to aphids and viruses they carry: from molecular structure to field effects. *Front. Plant Sci.* 7:1420. 10.3389/fpls.2016.01420 27725823 PMC5035753

[B14] Borowiak-Sobkowiak B. Woźniak A. Bednarski W. Formela M. Samardakiewicz S. Morkunas I. (2016). *Brachycorynella asparagi* (Mordv.) induced-oxidative stress and antioxidative defenses of *Asparagus officinalis* L. *Int. J. Mol. Sci.* 17:1740. 10.3390/ijms17101740 27775613 PMC5085768

[B15] Bos J. I. B. Prince D. Pitino M. Maffei M. E. Win J. Hogenhout S. A. (2010). A functional genomics approach identifies candidate effectors from the aphid species *Myzus persicae* (green peach aphid). *PLoS Genet.* 6:e1001216. 10.1371/journal.pgen.1001216 21124944 PMC2987835

[B16] Botha A.-M. van Eck L. Burger N. F. V. Swanevelder Z. H. (2014). Near-isogenic lines of *Triticum aestivum* with distinct modes of resistance exhibit dissimilar transcriptional regulation during *Diuraphis noxia* feeding. *Biol. Open* 3 1116–1126. 10.1242/bio.201410280 25361582 PMC4232770

[B17] Bright J. Desikan R. Hancock J. T. Weir I. S. Neill S. J. (2006). ABA-induced NO generation and stomatal closure in Arabidopsis are dependent on H_2_O_2_ synthesis. *Plant J.* 45 113–122. 10.1111/j.1365-313X.2005.02615.x 16367958

[B18] Burd J. D. Burton R. L. (1992). Characterization of plant damage caused by Russian wheat aphid (Homoptera: Aphididae). *J. Econ. Entomol.* 85 2017–2022. 10.1093/jee/85.5.2017

[B19] Carmody M. Waszczak C. Idänheimo N. Saarinen T. Kangasjärvi J. (2016). ROS signalling in a destabilised world: A molecular understanding of climate change. *J. Plant Physiol.* 203 69–83. 10.1016/j.jplph.2016.06.008 27364884

[B20] Carolan J. C. Caragea D. Reardon K. T. Mutti N. S. Dittmer N. Pappan K. (2011). Predicted effector molecules in the salivary secretome of the pea aphid (*Acyrthosiphon pisum*): a dual transcriptomic/proteomic approach. *J. Proteome Res.* 10 1505–1518. 10.1021/pr100881q 21226539

[B21] Carolan J. C. Fitzroy C. I. J. Ashton P. D. Douglas A. E. Wilkinson T. L. (2009). The secreted salivary proteome of the pea aphid *Acyrthosiphon pisum* characterised by mass spectrometry. *Proteomics* 9 2457–2467. 10.1002/pmic.200800692 19402045

[B22] Chaudhary R. Atamian H. S. Shen Z. Briggs S. P. Kaloshian I. (2014). GroEL from the endosymbiont *Buchnera aphidicola* betrays the aphid by triggering plant defense. *Proc. Natl. Acad. Sci. U.S.A.* 111 8919–8924. 10.1073/pnas.1407687111 24927572 PMC4066539

[B23] Chen X. Zhang Z. Vosman B. Broekgaarden C. (2014). Constitutive overexpression of the pollen specific gene SKS13 in leaves reduces aphid performance on *Arabidopsis thaliana*. *BMC Plant Biol.* 14:17. 10.1186/s12870-014-0217-3 25267093 PMC4243735

[B24] Clements K. M. Sorenson C. E. Wiegmann B. M. Neese P. A. Roe R. M. (2000). Genetic, biochemical, and behavioral uniformity among populations of *Myzus nicotianae* and *Myzus persicae*. *Entomol. Exp. Appl*. 95 269–281. 10.1046/j.1570-7458.2000.00666.x

[B25] Czarnocka W. Karpiński S. (2018). Friend or foe? Reactive oxygen species production, scavenging and signaling in plant response to environmental stresses. *Free Radic. Biol. Med.* 122 4–20. 10.1016/j.freeradbiomed.2018.01.011 29331649

[B26] Czerniewicz P. Sytykiewicz H. Durak R. Borowiak-Sobkowiak B. Chrzanowski G. (2017). Role of phenolic compounds during antioxidative responses of winter triticale to aphid and beetle attack. *Plant Physiol. Biochem.* 118 529–540. 10.1016/j.plaphy.2017.07.024 28778044

[B27] Das K. Roychoudhury A. (2014). Reactive oxygen species (ROS) and response of antioxidants as ROS-scavengers during environmental stress in plants. *Front. Environ. Sci.* 2:53. 10.3389/fenvs.2014.00053

[B28] Delledonne M. Zeier J. Marocco A. Lamb C. (2001). Signal interactions between nitric oxide and reactive oxygen intermediates in the plant hypersensitive disease resistance response. *Proc. Natl. Acad. Sci. U.S.A.* 98 13454–13459. 10.1073/pnas.231178298 11606758 PMC60892

[B29] Demidchik V. (2015). Mechanisms of oxidative stress in plants: from classical chemistry to cell biology. *Environ. Exp. Bot.* 109 212–228. 10.1016/j.envexpbot.2014.06.021

[B30] Drurey C. Mathers C. T. Prince D. C. Wilson C. Caceres-Moreno C. Mugford S. T. (2019). Chemosensory proteins in the CSP4 clade evolved as plant immunity suppressors before two suborders of plant-feeding hemipteran insects diverged. *BioRxiv* 10.1101/173278

[B31] Durak R. Bednarski W. Formela-Luboiñska M. Woźniak A. Borowiak-Sobkowiak B. Durak T. (2019). Defense responses of *Thuja orientalis* to infestation of anholocyclic species aphid *Cinara tujafilina*. *J. Plant Physiol.* 232 160–170. 10.1016/j.jplph.2018.11.018 30537603

[B32] Elzinga D. A. De Vos M. Jander G. (2014). Suppression of plant defenses by a *Myzus persicae* (green peach aphid) salivary effector protein. *Mol. Plant Microbe Interact.* 27 747–756. 10.1094/MPMI-01-14-0018-R 24654979 PMC4170801

[B33] Farnese F. S. Menezes-Silva P. E. Gusman G. S. Oliveira J. A. (2016). When bad guys become good ones: the key role of reactive oxygen species and nitric oxide in the plant responses to abiotic stress. *Front. Plant Sci.* 7:471. 10.3389/fpls.2016.00471 27148300 PMC4828662

[B34] Fischer H. F. (2021). *The Influence of Singlet Oxygen and Loss of Function of Fatty Acid Desaturase 7 in the Chloroplast on Aphid Resistance in Arabidopsis thaliana.* [dissertation]. Fayetteville, AR: University of Arkansas.

[B35] Flors C. Nonell S. (2006). Light and singlet oxygen in plant defense against pathogens: phototoxic phenalenone phytoalexins. *Acc. Chem. Res*. 39 293–300. 10.1021/ar0402863 16700528

[B36] Foley R. C. Gleason C. A. Anderson J. P. Hamann T. Singh K. B. (2013). Genetic and genomic analysis of *Rhizoctonia solani* interactions with Arabidopsis; evidence of resistance mediated through NADPH oxidases. *PLoS One* 8:e56814. 10.1371/journal.pone.0056814 23451091 PMC3581538

[B37] Foyer C. H. Noctor G. (2016). Stress-triggered redox signalling: what’s in pROSpect? *Plant Cell Environ.* 39 951–964. 10.1111/pce.12621 26264148

[B38] Foyer C. H. Rasool B. Davey J. W. Hancock R. D. (2016). Cross-tolerance to biotic and abiotic stresses in plants: a focus on resistance to aphid infestation. *J. Exp. Bot.* 67 2025–2037. 10.1093/jxb/erw079 26936830

[B39] Gadjev I. Vanderauwera S. Gechev T. S. Laloi C. Minkov I. N. Shulaev V. (2006). Transcriptomic footprints disclose specificity of reactive oxygen species signaling in Arabidopsis. *Plant Physiol.* 141 436–445. 10.1104/pp.106.078717 16603662 PMC1475436

[B40] Glazebrook J. (2005). Contrasting mechanisms of defense against biotrophic and necrotrophic pathogens. *Annu. Rev. Phytopathol.* 43 205–227. 10.1146/annurev.phyto.43.040204.135923 16078883

[B41] Goggin F. L. Quisenberry S. S. Ni X. (2017). “Feeding Injury,” in *Aphids as Crop Pests*, 2nd Edn, eds van Emden H. M. Harrington R. (Oxfordshire: CABI), 303–322.

[B42] Goggin F. L. Williamson V. M. Ullman D. E. (2001). Variability in the response of *Macrosiphum euphorbiae* and *Myzus persicae* (Hemiptera: Aphididae) to the tomato resistance gene *Mi*. *Environ. Entomol.* 30 101–106. 10.1603/0046-225X-30.1.101 33044624

[B43] Guo H. Gu L. Liu F. Chen F. Ge F. Sun Y. (2019). Aphid-borne viral spread is enhanced by virus-induced accumulation of plant reactive oxygen species. *Plant Physiol.* 179 143–155. 10.1104/pp.18.00437 30381318 PMC6324229

[B44] Guo H. Zhang Y. Tong J. Ge P. Wang Q. Zhao Z. (2020). An aphid-secreted salivary protease activates plant defense in phloem. *Curr. Biol.* 30 4826.e–4836.e. 10.1016/j.cub.2020.09.020 33035482

[B45] Hemetsberger C. Herrberger C. Zechmann B. Hillmer M. Doehlemann G. (2012). The *Ustilago maydis* effector Pep1 suppresses plant immunity by inhibition of host peroxidase activity. *PLoS Pathog.* 8:e1002684. 10.1371/journal.ppat.1002684 22589719 PMC3349748

[B46] Hogenhout S. A. Bos J. I. (2011). Effector proteins that modulate plant–insect interactions. *Curr. Opin. Plant Biol.* 14 422–428. 10.1016/j.pbi.2011.05.003 21684190

[B47] Hou S. Liu Z. Shen H. Wu D. (2019). Damage-associated molecular pattern-triggered immunity in plants. *Front. Plant Sci.* 10:646. 10.3389/fpls.2019.00646 31191574 PMC6547358

[B48] Jaouannet M. Morris J. A. Hedley P. E. Bos J. I. B. (2015). Characterization of Arabidopsis transcriptional responses to different aphid species reveals genes that contribute to host susceptibility and non-host resistance. *PLoS Pathog.* 11:e1004918. 10.1371/journal.ppat.1004918 25993686 PMC4439036

[B49] Jiang Y. Miles P. W. (1993). Responses of a compatible lucerne variety to attack by spotted alfalfa aphid: changes in the redox balance in affected tissues. *Entomol. Exp. Appl.* 67 263–274. 10.1111/j.1570-7458.1993.tb01677.x

[B50] Jwa N.-S. Hwang B. K. (2017). Convergent evolution of pathogen effectors toward reactive oxygen species signaling networks in plants. *Front. Plant Sci.* 8:1687. 10.3389/fpls.2017.01687 29033963 PMC5627460

[B51] Kaloshian I. Walling L. L. (2005). Hemipterans as plant pathogens. *Annu. Rev. Phytopathol.* 43 491–521. 10.1146/annurev.phyto.43.040204.135944 16078893

[B52] Kerchev P. I. Fenton B. Foyer C. H. Hancock R. D. (2012). Infestation of potato (*Solanum tuberosum* L.) by the peach-potato aphid (*Myzus persicae* Sulzer) alters cellular redox status and is influenced by ascorbate. *Plant Cell Environ.* 35 430–440. 10.1111/j.1365-3040.2011.02395.x 21736590

[B53] Kmieć K. Kot I. (2021). Physiological response of *Populus nigra* ‘Italica’ to galling aphids feeding. *Plant Biol.* 23 675–679. 10.1111/plb.13265 33780123

[B54] Kmieć K. Rubinowska K. Golan K. (2018). *Tetraneura ulmi* (Hemiptera: Eriosomatinae) induces oxidative stress and alters antioxidant enzyme activities in elm leaves. *Environ. Entomol.* 47 840–847. 10.1093/ee/nvy055 29672728

[B55] Kot I. Kmieć K. (2020). Poplar tree response to feeding by the petiole gall aphid *Pemphigus spyrothecae* Pass. *Insects* 11:282. 10.3390/insects11050282 32380670 PMC7291223

[B56] Kuśnierczyk A. Winge P. Jørstad T. S. Troczyñska J. Rossiter J. T. Bones A. M. (2008). Towards global understanding of plant defence against aphids – timing and dynamics of early Arabidopsis defence responses to cabbage aphid (*Brevicoryne brassicae*) attack. *Plant Cell Environ.* 31 1097–1115. 10.1111/j.1365-3040.2008.01823.x 18433442

[B57] Kuźniak E. Kopczewski T. (2020). The chloroplast reactive oxygen species-redox system in plant immunity and disease. *Front. Plant Sci.* 11:572686. 10.3389/fpls.2020.572686 33281842 PMC7688986

[B58] Lamb C. Dixon R. A. (1997). The oxidative burst in plant disease resistance. *Annu. Rev. Plant. Physiol. Plant. Mol. Biol.* 48 251–275. 10.1146/annurev.arplant.48.1.251 15012264

[B59] Lehmann S. Serrano M. L’Haridon F. Tjamos S. E. Metraux J.-P. (2015). Reactive oxygen species and plant resistance to fungal pathogens. *Phytochemistry* 112 54–62. 10.1016/j.phytochem.2014.08.027 25264341

[B60] Lei J. Finlayson S. A. Salzman R. A. Shan L. Zhu-Salzman K. (2014). BOTRYTIS-INDUCED KINASE1 modulates Arabidopsis resistance to green peach aphids via PHYTOALEXIN DEFICIENT4. *Plant Physiol.* 165 1657–1670. 10.1104/pp.114.242206 24963070 PMC4119046

[B61] Li X. Zhang H. Tian L. Huang L. Liu S. Li D. (2015). Tomato SlRbohB, a member of the NADPH oxidase family, is required for disease resistance against *Botrytis cinerea* and tolerance to drought stress. *Front. Plant Sci.* 6:463. 10.3389/fpls.2015.00463 26157450 PMC4477072

[B62] Liu X. Williams C. E. Nemacheck J. A. Wang H. Subramanyam S. Zheng C. (2010). Reactive oxygen species are involved in plant defense against a gall midge. *Plant Physiol.* 152 985–999. 10.1104/pp.109.150656 19965963 PMC2815885

[B63] Łukasik I. Goławska S. (2019). Biochemical markers of oxidative stress in triticale seedlings exposed to cereal aphids. *Acta Biol. Crac. Ser. Bot.* 61 35–46. 10.24425/ABCSB.2019.127745

[B64] Łukasik I. Kornacka A. Goławska S. Sytykiewicz H. Sprawka I. Wójcicka A. (2017). Effects of *Acyrtosiphon pisum* (Harris) infestation on the hydrogen peroxide content and activity of antioxidant enzymes in Fabaceae plants. *Allelopathy J.* 40 143–150. 10.26651/2017-40-1-1073

[B65] Łukaszewicz S. Politycka B. Borowiak-Sobkowiak B. (2021). Effect of selenium on alleviating oxidative stress in pea leaves caused by pea aphid feeding. *J. Plant Protect. Res*. 61 83–94.

[B66] MacWilliams J. R. Chesnais Q. Nabity P. Mauck K. Kaloshian I. (2021). Cowpea aphid resistance in cowpea line CB77 functions primarily through antibiosis and eliminates phytotoxic symptoms of aphid feeding. *Res. Sq.* 2021:522914. 10.21203/rs.3.rs-522914/v1

[B67] Madhusudhan V. V. Miles P. W. (1998). Mobility of salivary components as a possible reason for differences in the responses of alfalfa to the spotted alfalfa aphid and pea aphid. *Entomol. Exp. Appl.* 86 25–39. 10.1046/j.1570-7458.1998.00262.x

[B68] Mai V. C. Bednarski W. Borowiak-Sobkowiak B. Wilkaniec B. Samardakiewicz S. Morkunas I. (2013). Oxidative stress in pea seedling leaves in response to *Acyrthosiphon pisum* infestation. *Phytochem.* 93 49–62. 10.1016/j.phytochem.2013.02.011 23566717

[B69] Mai V. C. Drzewiecka K. Jeleñ H. Narożna D. Ruciñska-Sobkowiak R. Kêsy J. (2014). Differential induction of *Pisum sativum* defense signaling molecules in response to pea aphid infestation. *Plant Sci.* 221–222 1–12. 10.1016/j.plantsci.2014.01.011 24656330

[B70] Mai V.-C. Nguyen B.-H. Nguyen D.-D. Nguyen L.-A.-V. (2017). *Nostoc calcicola* extract improved the antioxidative response of soybean to cowpea aphid. *Bot. Stud.* 58:55. 10.1186/s40529-017-0211-9 29185129 PMC5705527

[B71] Mai V. C. Tran N. T. Nguyen D. S. (2016). The involvement of peroxidases in soybean seedlings’ defense against infestation of cowpea aphid. *Arthropod Plant Interact.* 10 283–292. 10.1007/s11829-016-9424-1

[B72] Martinez, de Ilarduya O. M. Xie Q. Kaloshian I. (2003). Aphid-induced defense responses in *Mi-1*-mediated compatible and incompatible tomato interactions. *Mol. Plant Microbe Interact.* 16 699–708. 10.1094/MPMI.2003.16.8.699 12906114

[B73] Miles P. W. (1978). Redox reactions of Hemipterous saliva in plant issues. *Entomol. Exp. Appl.* 24 534–539. 10.1111/j.1570-7458.1978.tb02814.x

[B74] Miles P. W. (1989). “Specific responses and damage caused by Aphidoidae,” in *Aphids, Their Biology, Natural Enemies and Control*, (Amsterdam: Elsevier), 23–47.

[B75] Miles P. W. (1999). Aphid saliva. *Biol. Rev. Biol. Proc. Camb. Philos. Soc.* 74 41–85. 10.1111/j.1469-185X.1999.tb00181.x

[B76] Miller G. Schlauch K. Rachel T. Cortes D. Torres M. A. Shulaev V. (2009). The Plant NADPH oxidase RBOHD mediates rapid systemic signaling in response to diverse stimuli. *Sci. Signal.* 2 1–11. 10.1126/scisignal.2000448 19690331

[B77] Moloi M. J. Van Der Westhuizen A. J. (2006). The reactive oxygen species are involved in resistance responses of wheat to the Russian wheat aphid. *J. Plant Physiol.* 163 1118–1125. 10.1016/j.jplph.2005.07.014 17032617

[B78] Moloi M. J. van der Westhuizen A. J. (2014). Involvement of nitric oxide in the Russian wheat aphid resistance response of wheat. *Cereal Res. Commun.* 42 119–125. 10.1556/CRC.2013.0044

[B79] Moloi M. J. van der Westhuizen A. J. Jankielsohn A. (2014). Nitric oxide is an upstream signal involved in the multisignalling network during the Russian wheat aphid resistance response and its application enhances resistance. *Cereal Res. Commun.* 43 29–40. 10.1556/CRC.2014.0018

[B80] Nicholson S. J. Puterka G. J. (2014). Variation in the salivary proteomes of differentially virulent greenbug (*Schizaphis graminum* Rondani) biotypes. *J. Proteomics* 105 186–203. 10.1016/j.jprot.2013.12.005 24355481

[B81] Noctor G. Reichheld J.-P. Foyer C. H. (2016). ROS-related redox regulation and signaling in plants. *Semin. Cell Dev. Biol.* 80 3–12. 10.1016/j.semcdb.2017.07.013 28733165

[B82] O’Brien J. A. Daudi A. Butt V. S. Bolwell G. P. (2012). Reactive oxygen species and their role in plant defence and cell wall metabolism. *Planta* 236 765–779. 10.1007/s00425-012-1696-9 22767200

[B83] Ortega-Villasante C. Burén S. Blázquez-Castro A. Baron-Sola A. Hernandez L. E. (2018). Fluorescent in vivo imaging of reactive oxygen species and redox potential in plants. *Free Radic. Biol. Med.* 122 202–220. 10.1016/j.freeradbiomed.2018.04.005 29627452

[B84] Pant S. Huang Y. (2021). Elevated production of reactive oxygen species is related to host plant resistance to sugarcane aphid in sorghum. *Plant Signal. Behav.* 16 1849523. 10.1080/15592324.2020.1849523 33270502 PMC7849690

[B85] Peccoud J. Simon J.-C. von Dohlen C. Coeur d’acier A. Plantegenest M. (2010). Evolutionary history of aphid-plant associations and their role in aphid diversification. *C.R. Biol.* 333 474–487. 10.1016/j.crvi.2010.03.004 20541159

[B86] Pogány M. von Rad U. Grün S. Dongó A. Pintye A. Simoneau P. (2009). Dual roles of reactive oxygen species and NADPH oxidase RBOHD in an Arabidopsis-*Alternaria* pathosystem. *Plant Physiol.* 151 1459–1475. 10.1104/pp.109.141994 19726575 PMC2773049

[B87] Pointeau S. Ameline A. Laurans F. Sallé A. Rahbé Y. Bankhead-Dronnet S. (2012). Exceptional plant penetration and feeding upon cortical parenchyma cells by the woolly poplar aphid. *J. Insect Physiol.* 58 857–866. 10.1016/j.jinsphys.2012.03.008 22440739

[B88] Pollard D. G. (1973). Plant penetration by feeding aphids (Hemiptera, Aphidoidea): a review. *Bull. Entomol. Res.* 62 631–714. 10.1017/S0007485300005526

[B89] Prince D. C. Drurey C. Zipfel C. Hogenhout S. A. (2014). The Leucine-Rich Repeat Receptor-Like Kinase BRASSINOSTEROID INSENSITIVE1-ASSOCIATED KINASE1 and the Cytochrome P450 PHYTOALEXIN DEFICIENT3 contribute to innate immunity to aphids in Arabidopsis. *Plant Physiol.* 164 2207–2219. 10.1104/pp.114.235598 24586042 PMC3982773

[B90] Queval G. Hager J. Gakière B. Noctor G. (2008). Why are literature data for H_2_O_2_ contents so variable? A discussion of potential difficulties in the quantitative assay of leaf extracts. *J. Exp. Bot.* 59 135–146. 10.1093/jxb/erm193 18332224

[B91] Quisenberry S. S. Ni X. (2007). “Feeding Injury,” in *Aphids as Crop Pests*, eds van Emden H. M. Harrington R. 1*^st^* (Oxfordshire: CABI), 331–352.

[B92] Rao S. A. K. Carolan J. C. Wilkinson T. L. (2013). Proteomic profiling of cereal aphid saliva reveals both ubiquitous and adaptive secreted proteins. *PLoS One* 8:e57413. 10.1371/journal.pone.0057413 23460852 PMC3584018

[B93] Rashid M. H. O. Khan A. Hossain M. T. Chung Y. R. (2017). Induction of systemic resistance against aphids by endophytic *Bacillus velezensis* YC7010 via expressing PHYTOALEXIN DEFICIENT4 in Arabidopsis. *Front. Plant Sci.* 8:211. 10.3389/fpls.2017.00211 28261260 PMC5309228

[B94] Rasool B. Karpinska B. Pascual J. Kangasjärvi S. Foyer C. H. (2019). Catalase, glutathione, and protein phosphatase 2A-dependent organellar redox signalling regulate aphid fecundity under moderate and high irradiance. *Plant Cell Environ.* 43 209–222. 10.1111/pce.13669 31702837

[B95] Rasool B. McGowan J. Pastok D. Marcus S. E. Morris J. A. Verrall S. R. (2017). Redox control of aphid resistance through altered cell wall composition and nutritional quality. *Plant Physiol.* 175 259–271. 10.1104/pp.17.00625 28743764 PMC5580759

[B96] Ren G. Wang X. Chen D. Wang X. Liu X. (2014). Effects of aphids *Myzus persicae* on the changes of Ca^2+^ and H_2_O_2_ flux and enzyme activities in tobacco. *J. Plant Inter.* 9 883–888. 10.1080/17429145.2014.982221

[B97] Rossi F. R. Krapp A. R. Bisaro F. Maiale S. J. Carrillo N. (2017). Reactive oxygen species generated in chloroplasts contribute to tobacco leaf infection by the necrotrophic fungus *Botrytis cinerea*. *Plant J.* 92 761–773. 10.1111/tpj.13718 28906064

[B98] Rossi M. Goggin F. L. Milligan S. B. Kaloshian I. Ullman D. E. Williamson V. M. (1998). The nematode resistance gene *Mi* of tomato confers resistance against the potato aphid. *Proc. Natl. Acad. Sci. U S A* 95 9750–9754. 10.1073/pnas.95.17.9750 9707547 PMC21408

[B99] Ryan J. D. Morgham A. T. Richardson P. E. Johnson R. C. Mort A. J. Eikenbary R. D. (1990). “Greenbugs and wheat: a model system for the study of phytotoxic Homoptera,” in *Aphid–Plant Genotype Interactions*, eds Campbell R. K. Eikenbary R. D. (Amsterdam: Elsevier), 171–186.

[B100] Saheed S. A. Liu L. Jonsson L. Botha C. E. J. (2007). Xylem – as well as phloem – sustains severe damage due to feeding by the Russian wheat aphid. *S. Afr. J. Bot.* 73 593–599. 10.1016/j.sajb.2007.05.008

[B101] Segmüller N. Kokkelink L. Giesbert S. Odinius D. van Kan J. Tudzynski P. (2008). NADPH Oxidases are involved in differentiation and pathogenicity in *Botrytis cinerea*. *Mol. Plant Microbe Interact.* 21 808–819. 10.1094/MPMI-21-6-0808 18624644

[B102] Sewelam N. Jaspert N. Van Der Kelen K. Tognetti V. B. Schmitz J. Frerigmann H. (2014). Spatial H_2_O_2_ signaling specificity: H_2_O_2_ from chloroplasts and peroxisomes modulates the plant transcriptome differentially. *Mol. Plant* 7 1191–1210. 10.1093/mp/ssu070 24908268

[B103] Shao Y. Guo M. He X. Fan Q. Wang Z. Jia J. (2019). Constitutive H_2_O_2_ is involved in sorghum defense against aphids. *Braz. J. Bot.* 42 271–281. 10.1007/s40415-019-00525-2

[B104] Shapiguzov A. Vainonen J. Wrzaczek M. Kangasjärvi J. (2012). ROS-talk – how the apoplast, the chloroplast, and the nucleus get the message through. *Front. Plant Sci.* 3:292. 10.3389/fpls.2012.00292 23293644 PMC3530830

[B105] Sharma P. Jha A. B. Dubey R. S. Pessarakli M. (2012). Reactive oxygen species, oxidative damage, and antioxidative defense mechanism in plants under stressful conditions. *J. Bot.* 2012 e217037. 10.1155/2012/217037

[B106] Shoala T. Edwards M. G. Knight M. R. Gatehouse A. M. R. (2018). OXI1 kinase plays a key role in resistance of Arabidopsis towards aphids (*Myzus persicae*). *Transgenic Res.* 27 355–366. 10.1007/s11248-018-0078-x 29777502

[B107] Singh B. U. Padmaja P. G. Seetharama N. (2004). Biology and management of the sugarcane aphid, *Melanaphis sacchari* (Zehntner) (Homoptera: Aphididae), in sorghum: a review. *Crop Protect*. 23 739–755. 10.1016/j.cropro.2004.01.004

[B108] Stevens M. Lacomme C. (2017). “Transmission of Plant Viruses,” in *Aphids as Crop Pests*, 2nd Edn, eds van Emden H. M. Harrington R. (Oxfordshire: CABI), 323–347.

[B109] Sun M. Voorrips R. E. Vosman B. (2020). Aphid populations showing differential levels of virulence on *Capsicum* accessions. *Insect Sci.* 27 336–348. 10.1111/1744-7917.12648 30353689 PMC7379501

[B110] Sun Y. Xia X. L. Jiang J. F. Chen S. M. Chen F. D. Lv G. S. (2016). Salicylic acid-induced changes in physiological parameters and genes of the flavonoid biosynthesis pathway in *Artemisia vulgaris* and *Dendranthema nankingense* during aphid feeding. *Gen. Mol. Res.* 15:1. 10.4238/gmr.15017546 26909993

[B111] Sytykiewicz H. (2015). Transcriptional responses of catalase genes in maize seedlings exposed to cereal aphids’ herbivory. *Biochem. Syst. Ecol.* 60 131–142. 10.1016/j.bse.2015.04.015

[B112] Sytykiewicz H. (2016). Deciphering the role of NADPH oxidase in complex interactions between maize (*Zea mays* L.) genotypes and cereal aphids. *Biochem. Biophys. Res. Commun.* 476 90–95. 10.1016/j.bbrc.2016.05.050 27178208

[B113] Sytykiewicz H. Chrzanowski G. Czerniewicz P. Sprawka I. Łukasik I. (2014). Expression profiling of selected glutathione transferase genes in *Zea mays* (L.) seedlings infested with cereal aphids. *PLoS One* 9:e111863. 10.1371/journal.pone.0111863 25365518 PMC4218852

[B114] Thatcher L. F. Foley R. Casarotto H. J. Gao L.-L. Kamphuis L. G. Melser S. (2018). The Arabidopsis RNA Polymerase II Carboxyl Terminal Domain (CTD) Phosphatase-Like1 (CPL1) is a biotic stress susceptibility gene. *Sci. Rep.* 8 1–14. 10.1038/s41598-018-31837-0 30194343 PMC6128934

[B115] Tjallingii W. F. Esch T. H. (1993). Fine structure of aphid stylet routes in plant tissues in correlation with EPG signals. *Physiol. Entomol.* 18 317–328. 10.1111/j.1365-3032.1993.tb00604.x

[B116] Veselova S. V. Burkhanova G. F. Rumyantsev S. D. Blagova D. K. Maksimov I. V. (2019). Strains of *Bacillus* spp. regulate wheat resistance to Greenbug Aphid *Schizaphis graminum* Rond. *Appl. Biochem. Microbiol.* 55 41–47. 10.1134/S0003683819010186

[B117] Wada S. Cui S. Yoshida S. (2019). Reactive Oxygen Species (ROS) generation is indispensable for haustorium formation of the root parasitic plant *Striga hermonthica*. *Front. Plant Sci.* 10:328. 10.3389/fpls.2019.00328 30967886 PMC6438919

[B118] Wang Q. Yuan E. Ling X. Zhu-Salzman K. Guo H. Ge F. (2020). An aphid facultative symbiont suppresses plant defence by manipulating aphid gene expression in salivary glands. *Plant Cell Environ.* 43 2311–2322. 10.1111/pce.13836 32596816

[B119] Wang W. Chen D. Zhang X. Liu D. Cheng Y. Shen F. (2018). Role of plant respiratory burst oxidase homologs in stress responses. *Free Radic. Res.* 52 826–839. 10.1080/10715762.2018.1473572 29732902

[B120] Wang Y. Loake G. Chu C. (2013). Cross-talk of nitric oxide and reactive oxygen species in plant programed cell death. *Front. Plant Sci.* 4:314. 10.3389/fpls.2013.00314 23967004 PMC3744911

[B121] Waszczak C. Carmody M. Kangasjärvi J. (2018). Reactive oxygen species in plant signaling. *Annu. Rev. Plant. Biol.* 69 209–236. 10.1146/annurev-arplant-042817-040322 29489394

[B122] Woźniak A. Bednarski W. Dancewicz K. Gabryś B. Borowiak-Sobkowiak B. Bocianowski J. (2019). Oxidative stress links response to lead and *Acyrthosiphon pisum* in *Pisum sativum* L. *J. Plant Physiol.* 240:152996. 10.1016/j.jplph.2019.152996 31352020

[B123] Woźniak A. Formela M. Bilman P. Grześkiewicz K. Bednarski W. Marczak Ł (2017). The dynamics of the defense strategy of pea induced by exogenous nitric oxide in response to aphid infestation. *Int. J. Mol. Sci.* 18:E329. 10.3390/ijms18020329 28165429 PMC5343865

[B124] Xu J. Padilla C. S. Li J. Wickramanayake J. Fischer H. D. Goggin F. L. (2021). Redox responses of *Arabidopsis thaliana* to the green peach aphid, *Myzus persicae*. *Mol. Plant Pathol.* 2021:13054. 10.1111/mpp.13054 33829627 PMC8126190

[B125] Yang H. Mu J. Chen L. Feng J. Hu J. Li L. (2015). S-nitrosylation positively regulates ascorbate peroxidase activity during plant stress responses. *Plant Physiol.* 167 1604–1615. 10.1104/pp.114.255216 25667317 PMC4378166

[B126] Yao L. Yang B. Ma X. Wang S. Guan Z. Wang B. (2020). A genome-wide view of transcriptional responses during *Aphis glycines* infestation in soybean. *Int. J. Mol. Sci.* 21:5191. 10.3390/ijms21155191 32707968 PMC7432633

[B127] Zhang Y. Fu Y. Fan J. Li Q. Francis F. Chen J. (2019). Comparative transcriptome and histological analyses of wheat in response to phytotoxic aphid *Schizaphis graminum* and non-phytotoxic aphid *Sitobion avenae* feeding. *BMC Plant Biol.* 19:547. 10.1186/s12870-019-2148-5 31823722 PMC6902339

[B128] Zhang Z. Henderson C. Gurr S. J. (2004). *Blumeria graminis* secretes an extracellular catalase during infection of barley: potential role in suppression of host defence. *Mol. Plant Pathol.* 5 537–547. 10.1111/j.1364-3703.2004.00251.x 20565628

